# Learning Systems versus Future Everyday Domestic Life: A Designer’s Interpretation of Social Practice Imaginaries

**DOI:** 10.3389/frai.2021.707562

**Published:** 2021-07-30

**Authors:** Emilia Viaene, Lenneke Kuijer, Mathias Funk

**Affiliations:** Future Everyday Group, Department of Industrial Design, Eindhoven University of Technology, Eindhoven, Netherlands

**Keywords:** forecasting, design inquiry, smart home technologies, learning systems, everyday life, social practices, social practice imaginaries

## Abstract

Smart home technologies with the ability to learn over time promise to adjust their actions to inhabitants’ unique preferences and circumstances. For example, by learning to anticipate their routines. However, these promises show frictions with the reality of everyday life, which is characterized by its complexity and unpredictability. These systems and their design can thus benefit from meaningful ways of eliciting reflections on potential challenges for integrating learning systems into everyday domestic contexts, both for the inhabitants of the home as for the technologies and their designers. For example, is there a risk that inhabitants’ everyday lives will reshape to accommodate the learning system’s preference for predictability and measurability? To this end, in this paper we build a designer’s interpretation on the Social Practice Imaginaries method as developed by Strengers et al. to create a set of diverse, plausible imaginaries for the year 2030. As a basis for these imaginaries, we have selected three social practices in a domestic context: waking up, doing groceries, and heating/cooling the home. For each practice, we create one imaginary in which the inhabitants’ routine is flawlessly supported by the learning system and one that features everyday crises of that routine. The resulting social practice imaginaries are then viewed through the perspective of the inhabitant, the learning system, and the designer. In doing so, we aim to enable designers and design researchers to uncover a diverse and dynamic set of implications the integration of these systems in everyday life pose.

## 1 Introduction

The smart home market is growing at a rapid pace. This is exemplified by regular investments in this market by companies such as Amazon, with its recent acquisition of Ring (maker of internet-connected doorbells and cameras) or Google, with its investments in Nest (maker of smart thermostats, among other smart products) (Ali and Yusuf, 2018). Along with investment rates, adoption rates are also growing. Companies have been marketing smart home technologies (SHT) to mainstream consumers at increasingly declining price points. Whereas before, they had only been embraced by a small affluent and tech-savvy group of early adopters, they are now increasingly being implemented by a wider public (Ury et al., 2014). The presence of SHTs in the home can range from a single programmable smart light to a fully connected network of smart thermostats, security systems, cleaning appliances, and more. These SHTs, equipped with various kinds of sensors, have the ability to make decisions based on their specific context. For instance, smart thermostats’ sensors measure the room temperature to decide whether to switch the heating on.

Additionally, these products are often able to gather data about the households in which they are used. Through machine learning this data enables them to learn over time to adjust their actions to the specific preferences and routines of the households in which they are used (e.g., particular schedules and temperature preferences). Learning thermostats might thus, for example, autonomously adjust their heating pattern by inferring an occupancy profile through pattern recognition (Jung and Jazizadeh, 2019). SHTs and their implemented learning systems are often regarded to have the potential to enhance households’ future everyday lives by promoting their comfort, convenience, security and entertainment (Aldrich, 2003). Additionally, they have been looked at as offering possibilities to help solve energy-related issues including peak electricity demand, energy security and energy poverty (Fischer et al., 2013; Tirado Herrero et al., 2018; Strengers et al., 2019).

Learning systems are already being implemented in various smart home applications such as the Nest thermostat. The preference of these learning systems for predictability, however, shows frictions with the reality of everyday life, characterized by its complexity and unpredictability (Dourish and Bell, 2011). User studies observing the adoption of the Nest, for instance, report that the device often makes erroneous assumptions about the inhabitants’ intent, resulting in unwanted changes to the temperature schedule (Yang and Newman, 2013). These studies also indicate that the users themselves in turn do not understand how the device interprets user input to create a schedule and how it senses their movement and occupancy (Yang and Newman, 2013).

As user studies, such as the one by (Yang and Newman, 2013) indicate, most issues which have so far been identified through empirical research, are revealed in the communication and shared autonomy between the learning system and the inhabitant of the home. By bringing the perspective of the learning system and the inhabitant forward, “the designer” emerges as a third actor. Namely, decisions made in the development stage manifest during the use stage of the system, potentially leading to frictions between programmed behaviors and situated circumstances. For example, a smart thermostat such as the Nest may decide to heat a room autonomously “when appropriate”, yet the decision of what this “appropriate heating” is, has been made in the development stage (Kuijer and Giaccardi, 2018).

The quick spread of learning systems in SHTs, along with the potential frictions and issues related to learning systems in everyday life pose major challenges for designers of these systems. So far approaches to deal with these challenges have mostly been reactive–analyzing and addressing issues after these systems have entered everyday life. The question of how to pro-actively anticipate those issues in the future thus emerges. To explore how this might be done, we may draw on methods from futures studies. Forecasting methods have often been used as marketable resources which have become key to many organizations’ strategies, which shows that there is a huge commercial interest in “good futures” (Urry, 2016). For example, during the last decades several scholars have attempted to look at past and present trends and research in domestic technologies to be extrapolated to future directions for SHTs and related technologies (Aldrich, 2003; Chan et al., 2009; El-Hawary, 2014). Others have used backcasting and scenario building methods in which possible future scenarios are built in order to help anticipate and plan for desirable futures (Graham et al., 2013; Kishita et al., 2017). A final interesting example of forecasting methods, on which we will draw in this paper, are socio-technical imaginaries. The aim of this method is to expose the social implications of technological advancements and visions by depicting them in collectively imagined ways of social life (Jasanoff and Kim, 2009). These forecasts of possible futures enable a contribution to their anticipation and planning. Yet, it is important to keep in mind that the future is uncertain and forecasts often turn out to be incorrect (Strengers et al., 2019). These rather abstract, and sometimes utopian (Strengers, 2013) future scenarios might be at odds with the reality of everyday life–which, as indicated above, is often not as organized and uniform. These types of forecasts often do not reveal the types of issues that come with the integration of learning systems in a dynamic everyday domestic life.

Consequentially, the question arises how to effectively build and analyze dynamic visions of the future that help to gain deeper understanding of the implications of design decisions for both inhabitants and learning systems. Eventually, such a method can offer insights on the longer term effects of design decisions on everyday life. In order to help build this understanding, in this paper we look at possible future scenarios of common social practices in the home context. To build and analyze possible futures of social practices in the home context, we draw on the method of social practice imaginaries (SPIs), developed by Strengers et al., (2019), based on socio-technical imaginaries. This method combines socio-technical imaginaries with theories of social practices. In contrast to the aforementioned (often technologically centered) forecasting methods, social practice imaginaries pay specific attention to everyday life, highlighting its complexity (Strengers et al., 2019, p. 111). Additionally, “they (other, technologically centered methods) fail to account for the inevitability that everyday practices will have changed (in the future)” (Strengers et al., 2019, p. 111). In response, the social practice imaginaries method emphasizes the dynamic nature of everyday social practices, implying the inevitability that social practices will change in the future as they have in the past (Strengers et al., 2019, p. 111).

In this paper, we construct three social practice imaginaries from the individual perspectives of the three aforementioned actors (inhabitant, learning system and designer) in order to illustrate how the social practices are perceived and performed differently from their individual points of view. Through this adaptation of the social practice imaginaries’ method in this paper, we build an initial typology of the possible implications the integration of learning systems in dynamic everyday social practices in the home could pose. Through this method and initial typology we aim to enable designers and design researchers to better anticipate possible issues and frictions before, in the future, they occur in use.

## 2 Background

Before moving into the next chapters of this paper, we briefly elaborate a few key subjects and how they are understood in the context of this paper.

### 2.1 Social Practices

In this paper we use social practice theories as our theoretic perspective. Social practice theories take practices as the fundamental unit of analysis. They are used to describe how practices are interconnected in social worlds through relationships (Reckwitz, 2002a; Warde, 2005). The social practice perspective has the benefit of providing “an analytical and conceptual basis for defining the processes that order practice performances” (Mylan and Southerton, 2018, p. 1148). It also emphasizes how, rather than being the product of individualistic behaviors and choices, practices are rooted in material and institutional circumstances (Mylan and Southerton, 2018). Shove et al. (2012), for example, have built a theoretical structure for analyzing social practices that contains the following three overarching elements:

1. Materials: things, technologies, and bodies that enable practices to take place. Materials have a physical nature.
2. Competences: the skill, know-how, and technique. Competence can take the form of tangible knowledge (such as a recipe or an instruction sheet), but it can also take the form of oral and bodily knowledge that circulates and shifts as a practice evolves and becomes embedded in a network.
3. Meaning: symbolic meanings, ideas and aspirations. Meaning is constantly modified and leads to the classification and understanding of practices and why they are carried out.

Shove et al. (2012, p. 14) contend that “social practices emerge, persist, shift and disappear when connections between elements of these three types are made, sustained and broken”. Practices can arise and grow as a result of a number of minor changes in everyday life, but they are facilitated by the introduction of new types of material, meaning, and competence. According to Shove et al. (2012), not all practices are treated equally or given an equal position in the everyday activity space. Rather, everyday life revolves around a small number of practices that demand specific quantities of time and energy. This leads to the contention by Shove et al. (2012, p. 41) that “configurations that work (i.e., practices) do so because material elements and those of meaning and competence are linked together, and transformed, through the process of doing”. The elements of practices continue to evolve, they thus have a “career” of a certain duration which can be traced over time, and “people become carriers of a practice (that) …change(s) through related processes of recruitment, defection and reproduction” (Shove et al., 2012, p. 40).

#### 2.1.1 Social Practice Imaginaries

The method of Social Practice Imaginaries as developed by Strengers et al. (2019) serves as a basis on which we build in this paper. This method combines socio-technical imaginaries with theories of social practices. Dynamic social practices of which technologies form an integral part are used to envision and enact possible futures (Strengers et al., 2019, p. 110). With this method, Strengers et al. seek to envision possible future scenarios by basing them on past and present ethnographic research on everyday social practices and on secondary data about the practices’ evolution and change. Central to social practice imaginaries is the dynamic nature of everyday social practices and the inevitability that social practices will change (Strengers et al., 2019, p. 111). To this end, social practice imaginaries are always presented as open and prone to a continual transformation of the possibilities they pose.

To illustrate their approach, Strengers et al. (2019) use a “stay-at-home pets” scenario in the conceptualization of energy issues that the energy sector aims to overcome through smart technology deployments. Based on secondary data on pet care patterns and a decade of ethnographic study, they conclude that “the stay-at-home pets scenario proposes a future in which companion animals … live in thermally-regulated indoor spaces and engage in various types of electronic entertainment, even when their human companions are not at home” (p. 113). This scenario has implications for levels and patterns of energy demand that aren’t anticipated in the mainstream forecasting methods used by energy companies. They move on to stress that, like with any scenario, it is unknown whether or not this scenario will come true. It is only helpful “to prepare for and imagine different, plausible possibilities” (p. 113).

#### 2.1.2 Everyday Crises of Routine

In this paper we refer to possible unexpected situations of everyday use in relation to learning systems as everyday crises of routine, as described by Reckwitz (2002b). These situations of everyday “crises” are “situations that are in some way exceptional, non-standard, non-routine or non-mainstream” (Kuijer et al., 2017, p. 18). Reckwitz specifies them as “constellations of interpretative interdeterminacy and of the inadequacy of knowledge with which the [human] agent, carrying out a practice, is confronted in the face of a “situation” (2002b, p. 255). Next to the focus on the human agent as responsible for crises of routine as considered by Reckwitz, the artificial agent (in the form of a learning system) should also be considered in this context. For this we look at the co-performance perspective as described by Kuijer and Giaccardi (2018), in which artefacts (such as SHTs) that are capable of learning and performing social practices are placed next to people. Co-performance, from this perspective, recognizes the dynamic discrepancies in capabilities between humans and artefacts, as well as the inherent recursive relationship between design and use (Kuijer and Giaccardi, 2018).

In light of this paper, we interpret crises of routine as situations in which the learning system is unable to cater to the behavior and needs of the inhabitant when they divert from a “learnable” routine. Instead, in these situations everyday life shows unpredictabilities that could not be taken into account through learning the inhabitant’s routine. Examples of “messiness” may include spontaneous user decisions or exceptional events, such as extreme weather, pets, pests, other devices that are broken, infrastructural break-down such as internet failure, black-out, etc. This may in turn lead to irritations or challenges posed by the learning system for the inhabitant to deal with. More generally, we refer to the unrealistic nature of the (ideal) scenario that is anticipated in the design process. As illustrated previously through the user study by (Yang and Newman, 2013), learning systems are often unable to interpret the intent of the inhabitant correctly–which can in part be attributed to the anticipation of an ideal scenario acted out by perfectly routinized “users” as opposed to not-so-ideal, messy and unpredictable domestic life, containing a multitude of interconnected practices carried out by several inhabitants.

### 2.2 AI: Learning Systems

Smart home sensors can monitor and log environmental factors and human activities as well as power usage. These sensors could be: 1) device sensors that track activities such as turning on or off devices; 2) wearable sensors that can monitor vital signs, as well as the user’s location or activity; or 3) environmental sensors that can detect, for example, temperature, light, and occupancy (Liciotti et al., 2020). Consequentially, once such structured data are available, they can be analyzed and learned from: data records can be used to train machine learning models that promise to predict the inhabitants’ behavior based on historical data and in doing so gain situational awareness–that is, “understand” a user’s intentions and adjust parameters accordingly (Andrei Klubnikin, 2018). Subsequently, a smart automation system is intended to use these data patterns to provide inhabitants with the promise of increased comfort, energy savings, and improved security features.

In order to anticipate the inhabitants’ behavior, the “smart” home aims to recognize their actions and understand their behavioural patterns (Liciotti et al., 2020). Predicting user preferences and actions could for example be used to adjust temperature, heating or lighting conditions to suit their activities. Learning systems can gather sensor data through aforementioned sensors or they can learn inhabitants’ preferences through user-input (Snow et al., 2017). The smart home can become activity aware when sensor data is labeled using an activity recognition algorithm (Dahmen et al., 2017). Additionally, user recognition is aimed at identifying different inhabitants of the same household to profile their behavioral trends individually, or to allow for improved security (Zuo and De With, 2005; Singla et al., 2010).

However, as real-world everyday environments are dynamic and full of unknown factors, sensor data can be vague, noisy, and sparse (Dahmen et al., 2017; Liciotti et al., 2020). While the issues and limitations posed by these learning mechanisms often tend to be viewed as a challenge for data collection (Zuo and De With, 2005), the reality of an unpredictable “messy” everyday life begs for a more integrated approach. There are plenty of social factors that can complicate inhabitants’ relationship with SHTs, including social dynamics, expectations, and contextually specific factors (Snow et al., 2017). The idea that SHTs can make informed and autonomic choices on, for example, energy consumption in the home conflicts with the reality of a domestic “mess” that implies a lack of order in an otherwise ordered reality (Strengers, 2014).

## 3 Methodology

As mentioned above, the communication and shared autonomy between the learning system and the inhabitant expose the majority of issues that the integration of learning systems in everyday life raises (Yang and Newman, 2013). In this paper, we bring the perspectives of the inhabitant and the learning system together in the context of domestic social practices to explore the implications their disparities pose. Additionally, the designer is brought forward as a third party since decisions made in the design stage–often based on an “ideal” user scenario–can arise during the use stage of the learning products, exposing challenges for the designer. By constructing social practice imaginaries from the individual perspectives of these three actors (inhabitant, learning system, designer), we can illuminate how the social practices are viewed and enacted differently by each of them. By doing so, we are able to identify future challenges and perhaps even new opportunities for the design of learning systems in an everyday domestic context.

We thus aim to highlight implications the integration of learning systems in a domestic context poses by applying a future-oriented method. This lies in contrast to the majority of research in the area of SHTs, which is often empirical and reactive and thus based on the current technologies available on the market. We also expect to find issues that lie at a practice level, and thus beyond user-system interaction: for example, effects on levels of energy demand of home appliances and shifts of everyday practices towards artificial limitations such as measurability and quantifiability (Kuijer and Giaccardi, 2018). Additionally, by drawing on social practice theories as a unit of research, the zoomed-out view of practices-as-entity [“a temporally unfolding and spatially dispersed nexus of doings and sayings” (Schatzki, 1996, p. 89)] together with the detailed nature of practices-as-performance [“the performing of the doings and sayings which actualizes and sustains practices in the sense of nexuses” (Schatzki, 1996, p. 90)] can be useful to link the designer’s perspective to the longer term effects of design decisions on everyday life.

In accordance to (Strengers et al., 2019) method of social practice imaginaries, we seek to envision possible future scenarios in this paper by basing them on past, present and emerging future trends within everyday social practices in which technologies play an integral part. Additionally, we also centralize the inevitability that social practices will change in the future as they have in the past (Strengers et al., 2019, p. 111), in a large part due to the implementation of SHTs with the capability of learning over time. However, in contrast to the method as developed by Strengers et al. (2019) we have made the following three implementations: 1) we bring in the artefact and designer perspectives more explicitly, 2) we add learning system-specific selection criteria, and 3) we build a structured typology of implications based on the challenges and opportunities elicited by the imaginaries.

In this paper, we analyze three different everyday social practices in the home context. We set our social practice imaginaries in the year 2030. We do so since we imagine that by 2030, AI and learning systems would be widely present within the everyday social practices we carry out in our domestic environments. Yet the question still exists whether by then these learning systems will be able to adapt to the unpredictabilities of everyday domestic life, or whether the inhabitants of the home will have to adapt to the learning systems’ preference for predictability.

### 3.1 Selecting the Social Practices

In selecting these social practices, we have used five criteria as listed in Table 1: 1) the practices are prone to radical change, now or in the future; 2) they encompass a meaningful boundary around a complex of related practices; 3) learning systems can be involved; 4) the learning systems are exposed to the impact of everyday life’s unpredictability; and 5) the practices are present in a mundane household now and in the future. The first two of these criteria have been adopted from the methodology outline as described by Strengers et al. (2019, p. 111). These criteria pertain to selecting social practices that are eligible to the method of social practice imaginaries. The third and fourth criteria ensure a direct link between the selected social practices and the technologies studied in this paper, namely learning systems in smart home technologies. The final criterion has been added to create a focus on the mainstream user, looking for everyday and widespread challenges rather than, for now, niche issues.

**TABLE 1:** The selected social practices versus the selection criteria.

Criterium	Waking up	Grocery shopping	Heating and cooling the home
The practices are prone to radical change, now or in the future	People used to wake up by relying on their internal body clocks, the sun, servants, or church bells, nowadays many young people could not imagine being able to wake up in time for our daily duties without the help of an alarm clock	While people used to do their grocery shopping in various different specialized physical shops, now we are able to select and order all of our groceries online on one single web page without even leaving the house	While many people, owning modern heating and cooling systems, are now able to set the inside temperature of individual rooms to an exact amount of degrees, most people (in the past, and sometimes in e.g. more rural areas still) used to decide whether or not to put another log on the hearth in order to keep one room warm enough
They encompass a meaningful boundary around a complex of related practices	The practice of waking up starts the moment an alarm clock goes off and ends when the next practice is initiated	Selecting or detecting items to be bought, buying them—either in a physical store or online—and placing them in their designated place in the home	Can be described as a more peripheral, dispersed practice: Achieved across inhabitants through understanding and configuring the system, as they ‘set things up’
Learning systems can be involved	A learning algorithm could be implemented in the controls over the lights, the temperature, the blindsetc.	The learning system could for instance predict and carry out new purchases	The learning system could adjust the temperature to the inhabitants’ habits and preferences
The learning systems are exposed to the impact of everyday life’s unpredictability	May take place at very different times and places unexpectedly. For example: may be affected by illness, newborn baby, sleepover…	May vary greatly depending on (last-minute) plans and schedules, diets, impulses. For example: may be impacted by (unexpected) guests, temporary promos in the supermarket…	The desired inside temperature may vary greatly during different periods of time or based on different indoor activity. For example: may be impacted by indoor work-outs, illness…
The practices are present in a mundane household now and in the future	Caters to the basic need of sleep	Caters to the basic need of nutrition	Caters to the basic need of shelter

These criteria have led us to the selection of the following social practices as can be found in Table 1: 1) waking up, 2) grocery shopping, and 3) heating and cooling the home. Each of these practices enable many opportunities for learning machines to be involved. For instance: in waking up, a learning algorithm could be implemented in the controls over the lights, the temperature, the blinds, etc.; in grocery shopping, the learning system could for instance predict and carry out new purchases; and in heating and cooling the home the learning system could adjust the temperature to the inhabitants’ habits and preferences. Secondly, all of these practices can vary greatly from one generation to another, as well as from one culture to another. As these practices cater to rather basic needs (sleep, nutrition and shelter), they are quite omnipresent in a mundane day to day life. Accordingly, they are prone to many “crises of routine”. For instance: waking up may take place at very different times unexpectedly; grocery shopping is quite sensitive to unexpected factors such as a lack of time or an incomplete (or missing) grocery list; and the desired inside temperature may vary greatly during a heatwave.

### 3.2 Building the Social Practice Imaginaries

In order to set the stage for the social practice imaginaries, we start by defining and analyzing each social practice and its dynamics. First, where and when and how the social practice takes place is defined. For example, which sub-context(s) of a home the practice touches and whether it takes place on a weekday or in the weekend. Second, we briefly look at the historic evolution of the social practice (e.g., has the practice changed dramatically in the past, and if so, how?) and current changes in the practice due to emerging trends (e.g., food box subscriptions influencing how we do our groceries)? Finally, we look at how learning systems can play a role in the social practices and whether they could play a (bigger) role in the future of the year 2030. Important to note is that, due to the used sources to build the background for each practice, the modelled imaginaries in this paper are primarily based on western-based households living in urban areas.

As specified above, a central emphasis should be placed on the unpredictability of everyday life. To this end, we formulate two alternative imaginaries for every social practice: in one imaginary, the inhabitants’ routine is flawlessly supported by the learning system, and in the other, everyday crises of that routine are featured that cause friction in the automation of the future smart home. To set the stage, we start with the “routine”, “happy path” imaginary. This first imaginary follows from the preceding analysis of the social practice, extending the learning system’s integration into the practice. This “routine” imaginary is then rebuilt to include some form of crises of the routine. The goal of this “crises” imaginary is to trigger even more possibilities for crises and to elicit questions on how the imaginary could come about and which challenges this poses learning systems to be integrated into everyday domestic contexts, both for the inhabitants of the home and for the technologies and their designers. The “crises” imaginary represents ‘the tip of the iceberg’ of the issues, challenges and questions that it represents. Its goals is thus not to present an account of how we think these social practices and its integrated learning system will or should look like in the future, but to trigger critique and questions.

To create a nuanced understanding of how the performances of social practices might come about in the future, we explicitly formulate these imaginaries through the eyes of the inhabitants, the learning system and, if applicable, the designer. The perspective of the inhabitant is brought in through a short textual excerpt of their performance of the practice. The perspective of the learning system is brought in by listing the sequential actions carried out by the individual (smart) products related to this scenario starting with the (user) actions triggering these actions. This perspective also takes into account the possible previous user actions that have influenced the course of action taken by the learning system(s). Finally, the designer’s perspective emerges in between the learning system and the inhabitant in the “crises of routine” scenario. The designer’s perspective highlights discrepancies in the learning system and inhabitant’s perspectives and lists the designer’s possible reasoning resulting in a preferable course of action by the learning system in such case. These diverse perspectives enable a broader view on the implications of these social practice imaginaries.

By building these different imaginaries for three different specific and ubiquitous domestic social practices, we derive a typology of issues that the integration of learning systems in the home raises. This also enables us to tie these issues back to previous research on this topic if applicable.

### 3.3 Questioning the Social Practice Imaginaries

When looking at these social practice imaginaries, we aim to elicit challenges within and between its three actors (inhabitant, learning system and designer). In this step, it is of course interesting to look at the imaginaries from different points of view. To this end, we have asked four fellow design researchers with different areas of interest to critically look at these imaginaries from their expert point of view (see Table 2) and to share the questions, critique, ideas or opportunities they encounter. These researchers all deal with AI and connected systems in their own research within the field of industrial design, yet from different perspectives. The four interest areas of these researchers are the following: 1) design fiction and behavior change—making up a design fiction profile; 2) relationships between (immaterial or material) connected objects and people–forming an anthropologically centered profile; 3) HCI, crowdsourcing and social computing—which constitutes a more technical profile; and 4) interactional morality–forming an ethics-centered profile. As shown in Table 2, in this paper these researchers will be referred to as, respectively: 1) the design fiction expert, 2) the anthropological expert, 3) the technical expert, and 4) the ethics expert.

**TABLE 2:** The interviewed researchers and their areas of expertise.

Expert	Interest area
1. Design fiction expert	Design fiction and behavior change—making up a design fiction profile
2. Anthropological expert	Relationships between (immaterial or material) connected objects and people–forming an anthropologically centered profile
3. Technical expert	HCI, crowdsourcing and social computing–which constitutes a more technical profile
4. Ethics expert	Interactional morality–forming an ethics-centered profile

## 4 Social Practice Imaginaries

### 4.1 Waking up

As Shove et al. (2012) explain, it is through countless recurrent individual performances of a practice that the inter-dependencies between elements which constitute the practice-as-entity are sustained over time. Yet there are always local variations in performance by different practitioners. The social practice of waking up is one case in which this is very visible. Morning routines may vary from the moment the inhabitant is awakened–either by the aid of an alarm clock, or by the cries of a baby, the rising of the Sun, etc.–, whereupon some people are in the habit of dozing for minutes on end, while others get up at once. Additionally, the performances of the individual practitioner may vary depending on the time and space where the practice takes place. For instance, there may be a difference between waking up on a weekday versus in the weekend, wherein a weekday morning may be more planned out and time-dependent than a Saturday of Sunday morning. In this paper, we choose to position this social practice imaginary on a Monday, a weekday. For many people, this makes for a predictable routine for a learning system to learn from, whilst also being prone to possible unexpected changes such as a day off or a sick day, which possibly throws off its reasoning.

A morning routine may be defined as the sequence of practices occurring between waking up and leaving the home or starting work (Shove et al., 2012, p. 106). For this imaginary, we focus only on the practice of waking up, which starts the moment an alarm clock goes off and ends when the next practice is initiated. In this case we look at a Monday morning, with the practice starting at 7:30 AM when an alarm goes off and ending when the inhabitant has entered the next room, in this case the kitchen where breakfast can take place next.

At first glance, one might say that throughout western history, not much has changed in the practice of waking up. We start by waking up, either naturally or by the sound of an alarm clock. Then, we get out of bed and may or may not make up the bed, we turn on the lights and open the blinds and we move on to the next part of our morning routine. Yet, it is in the periphery around these actions that change occurs. For instance, before the invention of the mechanical alarm clock in 1787, waking up on time presented a challenge, leading most to rely on their internal body clocks, the Sun, servants, or church bells (Epstein and Kalleberg, 2006). Near the end of the 19th century, before widespread adoption of alarm clocks, in certain countries such as Britain and Ireland, waking up was even sold as a service by knocker-uppers. A knocker-upper would, upon receiving a couple of pennies, wake people up by banging on their doors or windows (Russo, 2016). During the 20th century, alarm clocks have been prone to innovations to make waking up a more pleasant experience, introducing radio alarm clocks and the infamous snooze-button (Russo, 2016). Today however, a classic alarm clock is more of a rarity. For many people, the mobile phone and later the smartphone have substituted the alarm clock in the form of alarm apps. Yet, the shift in the practice of waking up doesn’t end there. Nowadays, there’s an array of creative takes on alarm clocks, both physical or in the form of an app. For instance, there now are alarm clocks on the market that simulate the sunrise, project the time, and even alarm clocks that roll away and require you to catch them. Most of these are aimed at promoting healthy wake-up habits (usually leading users away from the snooze button) or at making waking up a more pleasant and natural experience. And finally, although in an experimental and early adopters’ phase, there is also a surge of smart waking devices. The leading example being Philips’ SmartSleep device, with lights controlling the user’s breathing, personalized wake-up sounds and lights and several head-mounted sensors registering the user’s sleeping rhythm and environment. Another growing smart product group with the ability of waking people up are the smart voice assistants such as Amazon Echo and Google Home. These smart assistants enable a whole new array of possibilities through their connection with other smart devices in the home. For instance, the morning alarm can trigger lights or heating to be turned on. Additionally, there has been a growing market for sensing systems monitoring and assessing sleep with the goal of improving its users’ understanding of sleeping patterns in order to improve the quality of sleep. Repeated observations, assessment of temporal patterns, and self-experimentation are made possible with these portable devices (Kelly et al., 2012). These devices might thus, in theory, use your pulse to verify your identity, measure your body temperature, and adjust your home’s lighting, heating and other devices accordingly. Consequentially, these trends set the stage for the integration of learning systems in the following social practice imaginary.

#### 4.1.1 Setting the Stage: Routine

**FIGURE 1:**
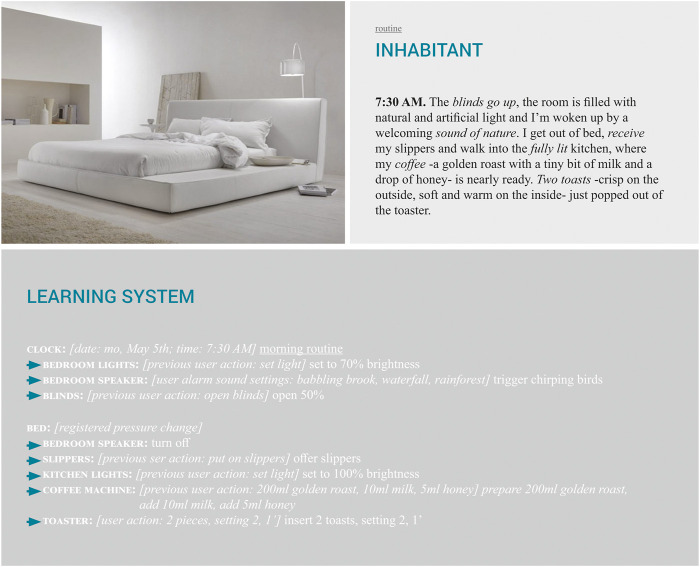
Getting up: routine imaginary (image by Vania, 2013).

Based on these historic developments and ongoing trends, the “routine” imaginary depicted in Figure 1 was built. This imaginary can be split into two parts: 1) in the first part, the inhabitant is woken up in a “natural” and calm way, reminiscent of smart waking devices such as Philips’ SmartSleep device; 2) after this, the inhabitant gets out of bed and walks into the next room, in this case the kitchen. Here, the next practice (having breakfast) is already triggered and prepared for the inhabitant.

#### 4.1.2 Food for Thought: Crises of Routine

**FIGURE 2:**
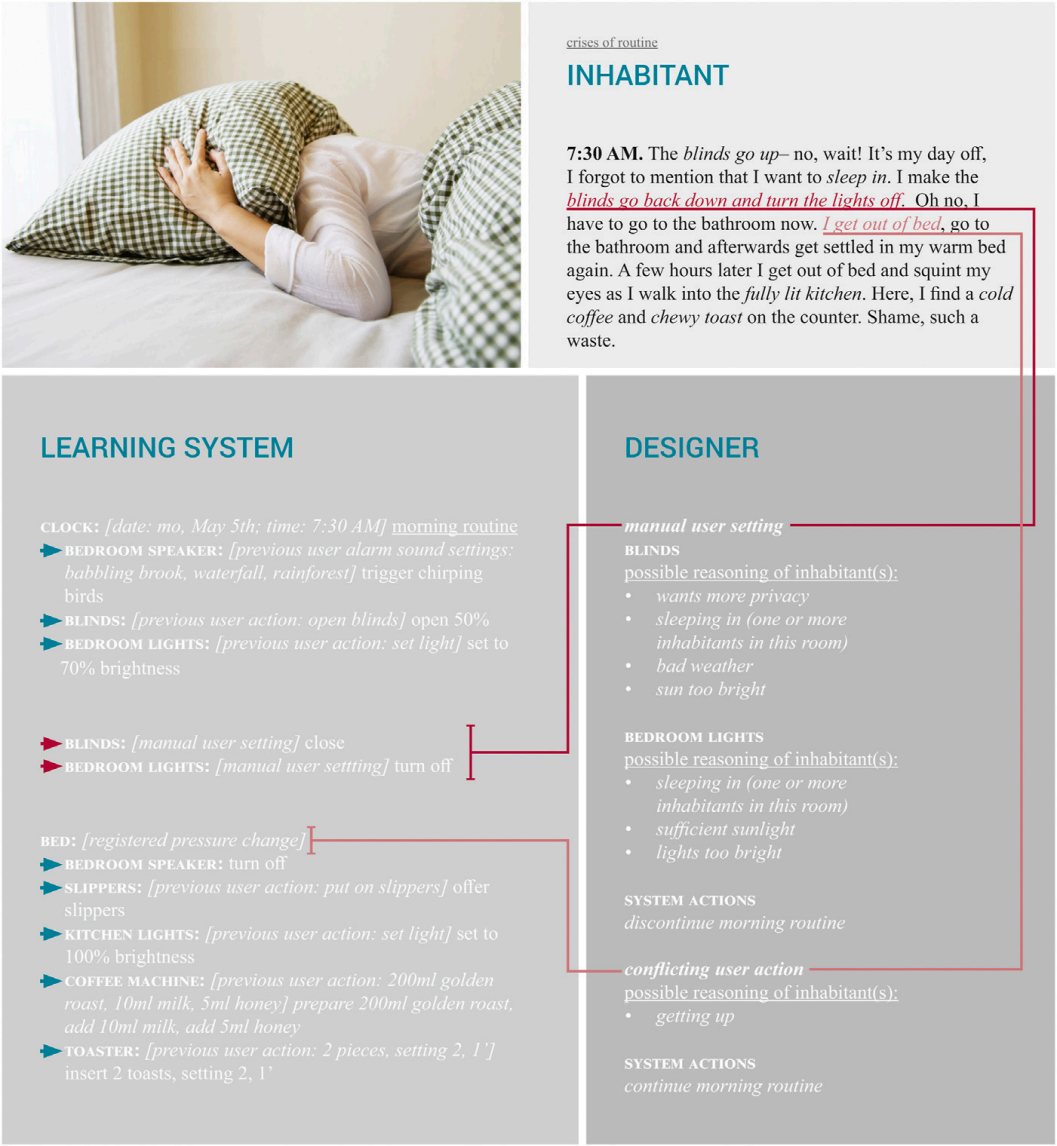
Getting up: crises imaginary (photo by Wuttichaikitcharoen, s.d.).

The “crises” imaginary in Figure 2 introduces two possible crises of routine in the experience of the inhabitant and the learning system, whilst also highlighting possible influences of the designers decisions. First, the inhabitant might have an unexpected day off work on which they want to sleep in. Yet the unaware learning system still triggers the morning routine, waking up the inhabitant. This could in turn lead to the inhabitant manually interfering by turning off the lights, closing the blinds and stopping the alarm while staying in bed. The designer’s influence could be to assess these types of situations as the inhabitant wanting to discontinue the morning routine, thus cancelling all the following system actions in the morning routine. A second crises could occur when, despite giving signals that they wish to sleep in, the inhabitant does get out of bed. In this case however, this is not because they want to get up, but because they go to the bathroom, after which they plan to get back in bed. The learning system (and the designer) however, might interpret this as the inhabitant deciding to continue their morning routine after all.

#### 4.1.3 Questions, Challenges and Opportunities

The “crises of routine” imaginary of the practice of waking up introduces only the tip of the iceberg, yet it enables us to speculate on possible other questions, challenges and opportunities. For instance, when looking at the second part of the imaginary, one of the first questions that arise is whether and how certain system actions require some form of preparations by the inhabitant. For instance, in order for the toaster to prepare two toasts, the slices of bread have to be inserted into the toaster by the inhabitant (or by a complementary system). Additionally, in this imaginary only one inhabitant is taken into account. This raises the question what could happen when a couple shares the bed. Or, in an even more irregular case, if for one night the bed is shared with another person who is not a known inhabitant of the home. The challenges this triggers is to account for multiple waking times and multiple morning routines overlapping and possibly influencing each other. For instance, can the system handle the preparation of multiple breakfasts at the same time or with just short intervals—and, does the risk exist that for example one breakfast is still waiting for the inhabitant and the next breakfast is already triggered, resulting in overflowing coffee cups or jammed toaster? And, in contrast what would happen if the inhabitant unexpectedly sleeps someplace else? Does the alarm still go off, are the lights still turned on, and will the blinds still be opened?

The design fiction expert raised some rather practical questions with this scenario. For instance, what happens when there is a power outage? This could raise issues with the connectivity and data processing of all of the connected systems. As illustrated with existing smart home technologies such as smart lighting systems, which could need to be reconnected again. Also, this expert questions whether the different systems influence each other when certain things occur. For instance, does the bedroom light’s brightness go up on a very dark and drowsy day in order to compensate for the missing light from outside? For the second part of the imaginary, the preparation of breakfast, she envisions that, if inhabitants’ are used to this degree of comfort, this might be pushed even further. In this light she wonders if for example, an inhabitant might only eat their toast with one particular spread—and what happens when 1 day they run out of this particular spread, does the toaster still prepare the toasts? On the same note, she wonders if the systems hold into account the different breakfast needs and wants at different times; for instance: one might not have a desire for the same breakfast when waking up at 4 AM as when waking up at 10 AM.

The technical expert questioned whether the system takes into account the sleep quality of the inhabitant, for instance through sensing systems integrated into the “smart” bed. For example, this could lead to the learning system adjusting the light and sound to the inhabitant’s sleep quality as well as taking into account when they are still in a deep sleep phase or in their REM-sleep. This also introduces a whole new level of feedback and communication between the learning system and the inhabitant. The learning system could perhaps learn and adjust their actions to factors which reside in the unconsciousness of the inhabitant. For instance, when the inhabitant is waking up in the morning, the lights may turn on slowly to anticipate the inhabitant getting out of bed, without the participant even having set an alarm.

The ethics expert approached this imaginary from a more pragmatic perspective, wondering what devices are and aren’t included in the learning system and what the responsibility of the inhabitant is in installing and connecting these devices. Since it is usually the inhabitants’ decision to implement these systems in their home, it would be preferred that they are also wary of the implications this has and that they adjust their expectations to the capabilities of the learning system. This raises the question whether it is desirable to expect the system to always know what you want or whether we should accept that these systems might not always be perfect, just like the people and pets you live with in the home. In this light, this expert also raises the risk of the system being hijacked by others not inhabiting the home. For instance, the system might be hacked by thieves to override security systems or even an ex-partner with access rights might misuse the system to give an inhabitant a hard time by for example driving up energy, gas or water bills, setting alarms at unholy times or flooding the bathroom.

The anthropological expert finally suggests that not all morning routines are equal and it might prove challenging to anticipate changing user preferences. For instance, on days where an inhabitant did not sleep well, they might prefer a stronger coffee. This might be inferred by the system through wearable sensors, but sometimes this might not be possible (e.g., in case of a spontaneous headache). Additionally, while some habits might be very constant (e.g., having toast for breakfast), others might be dependent on other factors over which the inhabitants themselves might not be in charge (e.g., dealing with a newborn baby).

### 4.2 Grocery Shopping

The practice of grocery shopping is one which can currently be carried out in vastly different ways by its practitioners. The “traditional” way to do grocery shopping still takes place in the physical supermarket–with or without a shopping list–, where one goes and buys all the groceries and checks out with the (physical) cashier. Alternative “new” ways of doing groceries can range from using a self-scanner to scan and pay for groceries in the supermarket; to selecting and ordering groceries online, which are then delivered at your doorstep or which are picked up at the supermarket; to independent food box subscriptions in which the costumer selects the recipes they wish to cook during the week and it’s ingredients are delivered at their home. Just as there are various ways of doing groceries, there are various and combined ways for practitioners to make use of them. For instance, one could make use of food box subscriptions, while still going to the supermarket to buy the items that are not included in the subscription; another might make use of online grocery services at the beginning of the week for their “big” groceries for the entire week, but can still hop into the supermarket during the week for “smaller” groceries when for example unexpectedly running out of one item. In addition, also the different household structures (single-person households or households with small children) and life phases (single life or family life) have a great influence on the needs and preconditions concerning grocery shopping (Berg and Henriksson, 2020).

For this imaginary, we define grocery shopping as selecting or detecting items to be bought, buying them—either in a physical store or online—and placing them in their designated place in the home (in the fridge, cupboard, cellar … ). To build the imaginary, we chose to account for weekly “big” groceries for a single-person household, in which items are selected that need to be ordered to last the coming week. This enables a possibly predictable routine for a learning system, while also being vulnerable to unexpected occurrences such as a dietary change, a last-minute holiday, a dinner party, etc.

As already mentioned, at present there are several alternative and “new” ways of doing groceries. Yet, when thinking of grocery shopping, the “traditional” way still takes place in a physical supermarket with a physical cashier. However, even this “traditional” supermarket has undergone many changes throughout history. Before modern supermarkets popped up at the start of the 20th century, people would go to farms, markets and separate shops for each category of food (Stanton, 2018). Corner stores were then the closest thing to what we now call a supermarket (Stanton, 2018). These corner stores were also often called “dry grocers” due to their stock not including fresh products such as meat, vegetables and bread, which were sold by specialized stores such as the butcher, the green grocer or the bakery (Stanton, 2018). In 1916, the first “self-service” supermarket, named “Piggly Wiggly”, opened their doors in Memphis, Tennessee in the United States (Ross, 2016). Before this, customers would hand over their grocery list to the clerk at the dry grocer who collected the items. In this “new” supermarket however, customers could choose from the products themselves which led companies to having to tempt the customer to buy their product, which also marks the origin of “branding” (Ross, 2016). The Piggly Wiggly supermarket also introduced shopping baskets, price-marked items, employees in uniform and the supermarket franchise model (Ross, 2016). After this, many other supermarkets popped up, with its success surging during the second world war (Ross, 2016). After the war, the rising popularity of refrigerators and automobiles kept feeding the model and as a result, free parking places became a necessity at every supermarket (Ross, 2016). As more and more technological advancements finally changed the way we do our groceries inside the supermarket during the decades following its introduction, changes have emerged outside the supermarket as well. Next to online grocery shopping, food box subscriptions such as Marley Spoon and Hello Fresh have made their way into people’s grocery habits. These technological trends also set the stage for the integration of learning systems for (online) grocery shopping at home as well as in the supermarket.

#### 4.2.1 Setting the Stage: Routine

**FIGURE 3:**
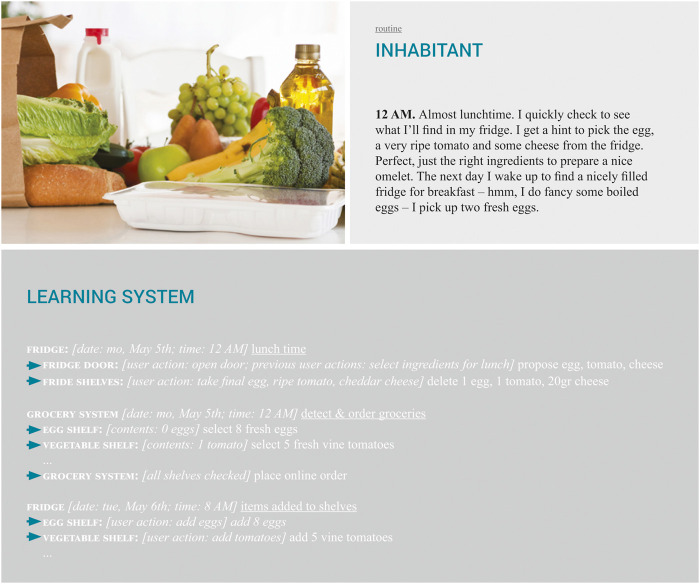
Groceries: routine imaginary (photo by Houston, s.d.).

These past, current and future trends lead us to the ‘routine’ imaginary, which is shown in Figure 3. In this imaginary, two grocery related situations take place, this imaginary can thus be split into two parts. In the first part, the inhabitant opens the fridge at noon, which in turn suggests some items with which the inhabitant could make their lunch. In the second part, the inhabitant, after waking up the next morning, finds that groceries have been done and new items have been added to the fridge. What happens in this imaginary from the perspective of the learning system is the following: 1) in the first part, the fridge infers from the fact that it is noon that the inhabitant will want to have lunch when opening its door; 2) in the second part, the grocery system has selected and ordered several items to be delivered and added to the fridge and other food shelves.

#### 4.2.2 Food for Thought: Crises of Routine

**FIGURE 4:**
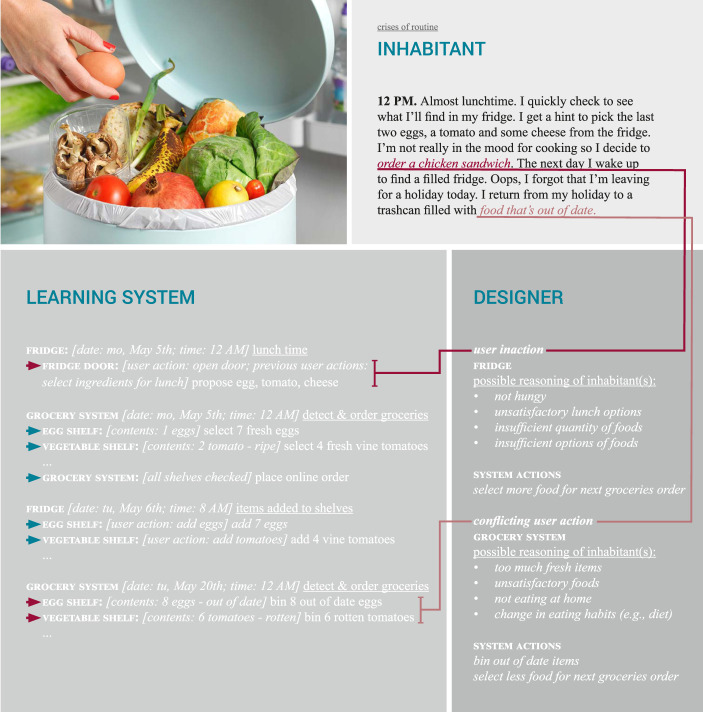
Groceries: crises imaginary (photo by Dazeley, s.d.).

The “crises” imaginary in Figure 4 introduces two possible crises of the everyday routine in each part of the imaginary. In the first part, the inhabitant does not comply with the suggestions of the fridge, but instead orders food from an external source. From the perspective of the designer, this could trigger the learning system to assess why the inhabitant has dismissed their suggestions (e.g., are they not hungry? are they not satisfied with the suggestion?). In turn this could lead to the learning system ordering or suggesting different, more or fewer food items in the future. In the second part, a crises of routine occurs when the inhabitant leaves for a holiday unexpectedly and without the learning system knowing about this. This could lead to the learning system carrying on with business as usual, and thus ordering new groceries. However, as the inhabitant is gone for a longer period of time, this leads to the food going out of date and being wasted as a result.

#### 4.2.3 Questions, Challenges and Opportunities

Looking at this “crises of routine” imaginary could again raise a multitude of related questions, challenges and opportunities. For instance, what happens when someone else—not an inhabitant of the home—or multiple people eat with me unexpectedly? Such situation might pose multiple implications: Does grabbing more food than necessary for the preparation of one lunch have effects on future actions of the learning system? Does the grocery system take into account this possibility, adjusting its orders to include more items for guests? etc. Additionally, one might wonder if the integration of ordering groceries would imply dealing with issues and errors from the grocery service such as replacing or removing items from the grocery list which are out of stock.

The design fiction expert raises the issue of inter-relatedness of the different practices. For example, if for whatever reason, there is no more bread, would this affect the triggering of breakfast preparations which implies preparing toast using slices of bread?

In the same light, the technical expert questioned what other factors could and should be taken into account by the learning system, since people base their eating behavior on more than what food is available and what time it is. For example, the mood of the inhabitant, which is a very subjective factor, can heavily influence their eating habits. Yet their mood might be affected by many (measurable) external factors such as the weather and their schedule. The question thus rises whether it would be beneficial and desirable if the learning system would take into account these contextual factors in order to make long-term considerations and understand the personality and context factors influencing the inhabitant’s behavior. This might also lead to embedding abnormal detection, identifying unexpected events and enabling the system to deal with unexpected scenario’s in a different way. Namely, if the learning system knows the routine practice, they may be aware of what is an unexpected situation. The learning system might however not be able to deal with all diverse situations, in case of a new unexpected event they may ask for feedback from the inhabitant. Which also begs the question of how the system would request input from the inhabitant, and how they would motivate the inhabitant not to ignore these feedback requests by the learning system.

This imaginary also triggered the ethics expert to take a more radical stance and question whether it is necessary or even desirable to have a learning system for preparing and ordering food all together. To illustrate, many people do not wish to plan what they will be eating the coming week, and instead decide on-the-spot when they have arrived in the supermarket. In addition, certain cultures would think of it as “wrong” to have fridge that tells them how to cook their foods, as it could take away the creative element of cooking. From the same perspective, this expert also questions whether this type of learning system would be used more as a means of giving suggestions to the inhabitant or whether it would be assumed that the system does the thinking for the inhabitant, dictating a certain routine. This line of thought leads to the question whether people who are not prone to live a “predictable” life would be inclined to buy such a (possibly expensive) system or that they would simply ignore or disable the learning system’s actions.

The anthropological expert questioned what type of assistance role taken on the learning system might be helpful versus unhelpful or even disruptive and annoying. Its virtue might however lie in managing food shortages for example. If this system could coordinate with larger systems of grocery suppliers, it could manage the continuous flow of supply instead of risking e.g., large buy-ins of a certain products (e.g., the high demand for toilet paper at the beginning of the pandemic). Thus eliminating possible human “irrational” decisions by buying into insights of a larger ecosystem of knowledge that an individual person could not have. Additionally, this expert argues that a learning system might also be good at trying to “hack” systems of e.g., evolving buying vs. eating habits. For example, when an inhabitant’s eating habits change, their buying habits might not have, yet a learning system might pick up on this possibly wasteful discrepancy in habits and act accordingly. Thus, a learning system might be useful when the agency as a user is removed in order to bridge the knowledge gap of the inhabitant.

### 4.3 Heating and Cooling the Home

In contrast to the practices of waking up and grocery shopping, heating and cooling the home can be described as a more peripheral, dispersed practice (Schatzki, 2010; Morley, 2016; Hampton, 2019). Morley (2016, p. 7) refers to the practice of “heating (and cooling) houses” as a practice which is not carried out by a single performance or practitioner at any one time. Rather, this practice is achieved across inhabitants through understanding and configuring the system, as they, as Schatzki describes (2010, p. 129) “set things up”. In this practice, “the concept of practices might be “extended” to include the operation of machines that share or take over the same tasks as human practitioners but which occur at some temporal or spatial distance from a range of human-enacted activities” (Morley, 2016). There is thus not one single practitioner of the practice of heating and cooling the home, nor are there single points in time in which this practice is carried out.

For purpose of building this imaginary however, we do consider a specific point in time in which the inhabitant carries out several actions possibly influencing the temperature of the home and exemplifying previous understanding and configuration of the system’s set-up. To build the imaginary, we thus look at time at which the inhabitant returns home from work and carries out tasks in the home influencing the temperature inside the home (e.g., opening a window). This enables an analysis of the possible influence of other practices, settings and materialities on the heating and cooling of the home at times of routine as well as at times of crises of this routine.

One of the main incentives for change in the practice of heating and cooling the home throughout history, and especially over the last century, has been to increase its energy efficiency (Isaac and Van Vuuren, 2008; Morley, 2016; Kuijer and Watson, 2017; Jung and Jazizadeh, 2019). This is not surprising, as heating, ventilation and air-conditioning (HVAC) systems account for more than half of the energy consumption in residential buildings (U.S. Energy Information Administration (EIA), 2020). The second main incentive for change is comfort. This, of course, conflicts with the incentive to use less energy in heating and cooling the house, which is why thermal comfort specification has been one of the most controversial topics in building science (Nicol and Humphreys, 2002; Chappells and Shove, 2007). Going back in time, we find that central hearths date back as far as 2500 B.C. (Nagengast, 2001). Since then, there has been a rich history of heating systems evolving from fireplaces to stoves to (Roman) underfloor heating systems to warm-air heating systems to steam heating to open coal fire heating systems and finally to gas central heating (Nagengast, 2001). In addition, homes have become more and more easy to heat as a result of improved thermal insulation. In contrast to heating the home, the practice of cooling the home has a much shorter history in terms of technological advancements. Its foundation was laid in the 1840s, with Dr. John Gorrie’s invention of a rudimentary system for cooling hospital rooms using ice that has been created by his machine which used a compressor powered by a horse, water, wind-driven sails or steam to turn water into ice (Lester, 2015). Then, in 1902 William Carrier created the first modern air-conditioning system (Lester, 2015). These historic advancements in heating and cooling systems mark the start of a change in what people take to be appropriate, “natural” and necessary indoor thermal conditions (Chappells and Shove, 2007). There seems to be an unstoppable demand for air conditioning (and central heating) in one form or another, as well as the standardized conditions it enables (Chappells and Shove, 2007; Building Services Research and Information Association, 2017). As a result, it appears that reproducing comfort is becoming a more and more resource-intensive practice (Chappells and Shove, 2007).

Over the past decade, studies have shown that heating, ventilation, and air-conditioning (HVAC) systems have a number of major shortcomings such as conditioning unoccupied spaces, assuming maximum occupancy in spaces, and over-conditioning in buildings regardless of occupants’ perspectives (Erickson et al., 2009; Erickson and Cerpa, 2010). The primary cause of this is the fact that HVAC systems in their conventional operating modes (through thermostats with fixed operating schedules and single-point temperature measurement) do not account for occupant dynamics (Jung and Jazizadeh, 2019). In tackling these shortcomings, advances in ICT have made it possible to collect and communicate data in real time, as well as to implement data-driven pattern recognition and control algorithms (Jung and Jazizadeh, 2019). As a result, research has shifted to Human-In-The-Loop (HITL) operations, which rely on data from human experiences, such as accounting for occupant dynamics in indoor environments (e.g., occupancy-related features such as presence, count, and position, and thermal comfort) (Snow et al., 2017; Jung and Jazizadeh, 2019). Through future developments, HVAC systems are expected to be aware of actual occupancy schedules, plan preferred conditions prior to occupant arrival, maintain them during occupancy, and change operations once the vacancy is verified to reduce energy waste (Jung and Jazizadeh, 2019). As such, it is clear that the integration of learning systems in the social practice of heating and cooling the home is becoming a reality at a fast pace (and already is: e.g., Nest).

#### 4.3.1 Setting the Stage: Routine

**FIGURE 5:**
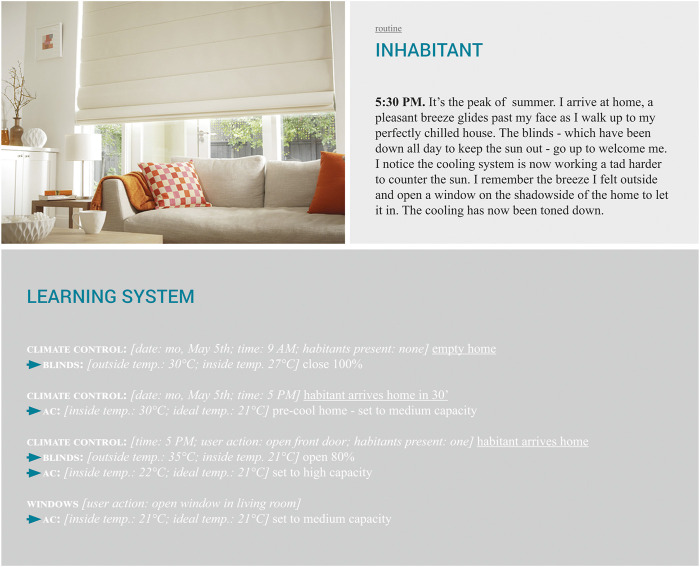
Heating and cooling: routine imaginary (photo by Bunnings, s.d.).

Based on these developments throughout history and across ongoing and future trends, the “routine” imaginary for heating and cooling the home as depicted in Figure 5 was built. In this imaginary, the stage is set by an excerpt of a performance by the inhabitant. The arrival of the inhabitant on a hot summer day has been anticipated by the learning system, which makes sure they come home to a perfectly chilled home. We note that in order to counter the Sun, the blinds had been down prior to their arrival. Finally, when opening a window to let in a cool breeze, the system reacts by toning down the air-conditioning.

#### 4.3.2 Food for Thought: Crises of Routine

**FIGURE 6:**
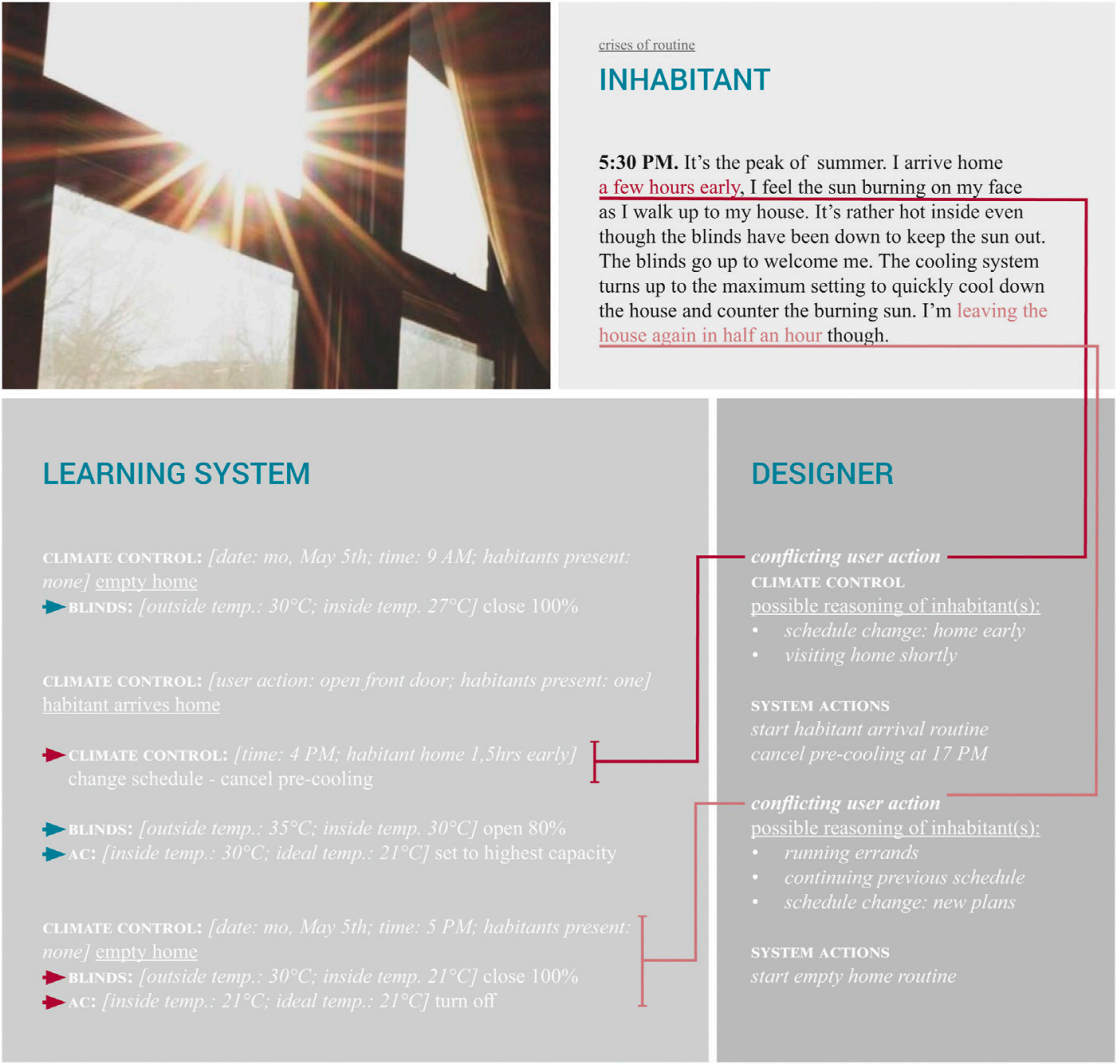
Heating and cooling: crises imaginary (photo by Vermeersch, s.d.).

In the imaginary shown in Figure 6, two possible crises of routine are introduced. The first crises occurs when the inhabitant arrives earlier than could be anticipated by the learning system. This leads the system to cancel the pre-cooling scheduled later that day and to start working to cool the home as quickly as possible. However, as the house is working hard to cool down the home, the inhabitant will be leaving the house again in only 5 min. With this second crises, again a decision has to be made on whether or not to turn off the air-conditioning and close the blinds.

#### 4.3.3 Questions, Challenges and Opportunities

On first glance, this imaginary can elicit several questions pertaining to the occupancy of the home. For instance, in order to better anticipate occupancy, the learning system might infer that an inhabitant will be at home or arriving home by learning their schedules and possibly tracking their smartphone. Yet, does the system also take into account pets and other inhabitants of the home such as a child? Additionally, does the risk exist that the system lock these types of inhabitants in or out of the home by closing the blinds when no occupancy is detected or assessed? Another issue that immediately surfaces is energy savings. Namely, is it desirable for the system to anticipate and react every time an inhabitant enters the home, creating the “ideal” atmosphere, even if the inhabitant might only be inside for a very limited amount of time?

In this imaginary, the technical expert sees an opportunity for tying in external factors to feed the learning system responsible for heating and cooling the home in order to anticipate certain events. For example, through information gained on traffic they could anticipate the inhabitant being home earlier or later depending on whether or not there might be traffic jams. Additionally, by tying this system to other practices such as waking up they might anticipate when the inhabitant will wake up and adjust the temperature accordingly.

Another factor raised by the design fiction expert is the care of houseplants. What happens to plants that are sensitive to temperatures and humidity, does the learning system take these factors into account, allowing for the right humidity, sunlight, etc.? And perhaps more importantly, would this be a desirable course of action or could this be perceived as wasteful as it would once again take up energy and actions which might not be necessary or even desirable for the inhabitants of the home. Another question raised by this expert is how such a system might take into account different preferences and habits for different inhabitants of the same home. For example, one inhabitant could prefer warmer and another cooler temperatures in the same room.

The ethics expert again raises the issue of possible hijackings of the system in this imaginary. Namely, a person meaning to harm or annoy the inhabitant(s) could turn up or down the temperature to unbearable conditions, whilst also spicing up the energy bills and posing possibly environmentally harmful behavior. This expert also raises the opportunity for these learning systems to take into account contextual factors such as weather forecasts in order to anticipate and suggest certain behaviors. For example, during a heatwave the system could suggest opening or closing the windows at certain times in order to avoid overly cooling the home through AC systems. Additionally, it might be beneficial to consider the background of people. For example, in areas suffering from particularly high temperatures, such as Texas, people currently tend to make very heavy use of AC systems. They have the lived experience of dealing with these systems without the learning systems and might or might not benefit from its suggestions and learned actions.

## 5 A Typology of Identified Implications

As illustrated in the sections above, the imaginaries enable the elicitation of a multitude of critical questions, challenges and opportunities for the integration of learning systems in an everyday domestic context. However, since these questions, challenges and opportunities can be regarded from very different perspectives, it could prove challenging to navigate these fields of reflection. To this end we have structured them into a typology in Table 3. This typology indicates the different types of reflections that might be elicited through building “routine” and “crises of routine” imaginaries of common social practices. The different types of implications are illustrated with a short example from the three different imaginaries in this paper.

**TABLE 3:** Typology of identified implications.

Type of implication	Examples from social practice imaginaries critique
**1. Technological limitations**	
These limitations refer to the practical, technical factors underlying the practices carried out by the learning systems. In many cases, the system will require some kind of interference by the inhabitant in order to be able to carry out their actions. On the other hand, these systems are also part of a larger network of system (e.g., power network, WiFI-network, the cloud, other IoT devices, etc.), implying their reliance on these networks, resulting in possible chains of issues when an issue occurs somewhere in this network.	
Preparations	Grocery shopping: Placing the groceries in their designated places after they arrived at the inhabitants’ doorstep
Hardware failures	Waking up: The configuration of the lights, blinds, coffee machine etc. could be disrupted when there is a power outage
**2. Communication**	
The implications concerning the communication encompass the abilities of the learning system to communicate with their surroundings in the wide sense. Communication in this sense refers not only to the interaction between the system and the different inhabitants, but also to the links with the wider institutions of which the practice may be part ánd to the communication between multiple learning systems that encompass multiple diverse domestic practices.	
Feedback	Heating and cooling: In case of a new unexpected event the system may ask for feedback from the inhabitant. But would they motivate the inhabitant not to ignore these feedback requests by the learning system?
Learning opportunities	Grocery shopping: If this system could coordinate with larger systems of grocery suppliers, it could manage the continuous flow of supply instead of risking e.g. large buy-ins of a certain products
Complexes of practices	Grocery shopping: If there is no more bread, would this affect the triggering of breakfast preparations which implies preparing toast using slices of bread?
**3. Agency**	
The agency implications concern factors dealing with the learning systems’ interference with the inhabitants’ (sense of) control and maintenance over certain domestic practices. These factors include the negotiation over agency in practices, the systems’ dealing with the multiple–sometimes unexpected–inhabitants involved, and the bounds and limits of the systems’ responsibilities and (un)desirable actions.	
Division of agency	Grocery shopping: Certain cultures would think of it as ‘wrong’ to have system that tells them how to cook their foods, as it could take away the creative element of cooking
Usees	Waking up: What could happen when a couple shares the bed? Or, in an even more extreme and unexpected case, if for one night the bed is shared with another person who is not a known inhabitant of the home
(Un)desirable Behavior	Heating and cooling: Is it desirable for the system to anticipate and react every time an inhabitant enters the home, creating the ‘ideal’ atmosphere, even if the inhabitant might only be inside for a very limited amount of time?
**4. Data usage**	
The fact that the learning systems deal with data, both in feeding and controlling their actions, leads to several implications. In the imaginaries, two types can be identified. On the one hand, the issue of privacy occurs in sharing the data with thirds, aimed at catering to the inhabitants’ needs and wants, whilst also possibly leaking into other needs and wants by the institutions with whom the data is shared. On the other hand, the connectedness of the home and the importance of the data in maintaining and controlling the home, may lead to issues of safety, since this data, in the wrong hands, could lead to harmful practices.	
Privacy	Grocery shopping: Coordinating with larger systems of grocery suppliers, enabling the system to continually suggest promotions, new products … based on the inhabitants’ personal eating habits
Safety	Waking up: A person meaning to harm or annoy the inhabitant(s) could hijack the system and turn up or down the temperature to unbearable conditions, whilst also spicing up the energy bills and posing possibly environmentally harmful behavior

It is important to stress that we recognize that we are not the first to come across these implications. For example, looking at the technical limitations (preparations, more specifically), Fink et al., 2013 describe that most households making use of a vacuum cleaning robot would make adjustments to the space it is used in by e.g., putting cables away or pushing chairs aside.

Several implications listed in this typology also draw a line to Pierce’s work, in which he describes the “lines of creepiness” (Pierce, 2019). For instance, his concept of “digital leakage” corresponds with the instances identified through our imaginaries, categorized here as “data usage” implications, in which the use of data creates windows for external parties to share, steal and misuse it “in ways unbeknownst and possibly harmful to those to whom the data pertains, originates, or belongs” (Pierce, 2019, p. 3). His concept of “hole-and-corner applications”, on the other hand, links certain issues pertaining to data usage as well as to agency. Namely, the presence of hidden applications in devices exposes possibilities for the learning system, unbeknownst to and quite possibly unwanted by the inhabitants. In doing so, the inhabitants lose part of their agency in this process, whilst they might also unknowingly and unwillingly be sharing their data. As Pierce points out, these devices might be gathering an immense amount of intimate data that hold great value to greater organizations and institutions (Pierce, 2019, p. 8). Though the linking of these two implications, a third catches our attention. Namely, when looking at the implications pertaining to the communication, we notice that several of them point out possibilities in dealing with unpredictabilities through connecting inhabitants, learning systems, and wider institutions and organizations. Yet, these possibilities, holding up a positive tone, can be placed across the other implications, such as those pertaining to data usage and agency. By doing so, we realize that behind these possibilities, risks are hiding, possibly leading to loss of autonomy and data leakage.

In conclusion, many of these implications–if not all–have been individually analyzed and elaborated in previous research by various researchers within different fields of expertise. The strength of the method and its derivation of a typology, however, does not lie in reinventing the wheel, but in recognizing and bundling an array of specific implications in specific domestic practices. Analyzing these typologies holds the potential to shed light on different questions, possibilities and challenges from different and possibly unexpected angles. Through structuring and analyzing the typologies, learning systems’ designers can be thus be better equipped to anticipate and deal with the specific implications encountered when introducing a learning system in a certain “messy” domestic social practice.

## 6 Discussion

This paper has led us to reflect on plausible futures for learning systems in the future Everyday at home. However, the approach and its outcomes also elicit some higher-level points of discussion. The questions arising can lead us to reflect on our expectations of future application of these learning systems as well as on to what extent inhabitants are willing to delegate certain aspects of their daily life to the learning system. In this final section, we touch on three questions we have come across in this paper. The first question deals with the role of the learning system. Within this question we reflect on the distinction between the unpredictable everyday and its (multiple) inhabitants’ conflicting expectations versus the learning system’s capabilities and preference toward predictable routines. This distinction consequentially leads to the second question, focusing on the scoping of the agency divided between the inhabitant and the learning system. Here, we question whether we can expect the system to be able to fully adapt to its inhabitants or whether it would become expected for the inhabitants—choosing to implement learning systems in their home–to adapt to the learning systems capabilities. Finally, the latter questions lead to the questioning of everyday crises as a starting point and their potential to identify sources of longer term change.

The first of these questions deals with the role of the learning system. The typology of implications raised through this approach not only leads to several practical considerations for the designers–such as the need for human preparations, or the consideration of privacy and safety when using inhabitants’ data–, but also elicits the question of what role a learning system should or should not fulfill given the complex and unpredictable Everyday. Namely, is the definition of a perfect system one that is able to predict the unpredictable? And is this a desirable, or even realistic, expectation to have? Instead, designers could consider that sometimes a learning system is simply not beneficial or even welcome in domestic everyday life. For instance, when considering implications of agency, it is difficult and perhaps not even ideal to account for the needs and wants of every inhabitant possibly involved in a practice. Instead, it might be worthwhile to take a more nuanced perspective: learning systems have narrow properties that allow them to take control in some practices, but fall short in others. Learning systems are, for example, uniquely suited to detect long-term changes and patterns in our behavior–often better than we are–, which can be used to automate aspects of daily practices. To illustrate the latter, a system can maintain repeated grocery orders of items as long as they are consumed consistently and repeatedly, and adapt or discontinue the ordering automation as soon as the inhabitants no longer (routinely) consume them. In doing so, such a system could help limit the households’ food waste and grocery expenses. Yet, in other cases, inhabitants prefer or even depend in a higher degree on variety and spontaneity. For example, people across cultures greatly value the creative aspects of cooking, with deliberate improvisation and overstepping of rules and conventions when preparing meals.

The second question deals with the scoping of agency. The question that might be raised here is whether it is desirable to expect the system to always know what inhabitants want or need, or whether we should accept and design for the reality that these systems might fail, just like the people and pets we live with at home. For instance, when considering implications of communication, agency, and technological limitations, we might refer to the fact that it is usually the inhabitants’ decision to implement the learning systems in their home in the first place. Is it then not preferably expected from them to be wary of the learning systems’ limitations and to adjust their expectations to their capabilities? In other words, to what extent is it the learning systems’ responsibility to deal with the unpredictability of domestic everyday life, rather than it being expected from the inhabitants–choosing to live with a learning system–to adapt to the learning systems’ preference for predictability?

Finally, the use of everyday crises as a starting point is questioned. The questions above might be exceeding the idea of everyday crises of routine as a starting point, referring to a bigger picture of implementing learning systems in everyday domestic life. This in turn begs the question whether crises of routine are best suited to identify sources of longer term change. However, we argue that they are, simply because it is by zooming out from the countless plausible everyday crises that we recognize these greater questions. In turn, it is through everyday crises of routine that modifications of the practices might be considered, either through the inhabitants dynamically adapting to the learning systems’ sources of everyday crises or vice-versa.

## 7 Conclusion

In this paper we have drawn on methods from future studies to address the challenge of proactively anticipating issues related to the integration of learning systems in the complex and unpredictable everyday domestic life. We have based our approach on social practice imaginaries as developed by Strengers et al. (2019), centralizing the dynamic nature of social practices. Yet in order to cater to the specific context of SHTs with some form of learning integrated, we have set our approach apart from the one by Strengers et al. through the following three implementations: 1) we have added three learning system-specific selection criteria; 2) we have applied a designerly interpretation of the method by bringing in the designer’s and learning system’s perspectives explicitly next to the inhabitant’s perspective; 3) we have built and analyzed a structured typology of implications based on the challenges and opportunities elicited by the imaginaries.

The aim of the approach set forth in this paper is to enable designers and design researchers to elicit diverse critical reflections, highlighting (sets of) implications that might otherwise not be considered. While the typology we have arrived to in this paper depicts diverse implications that have been used in critical reflection, we acknowledge that this list is not exhaustive and conclusive. By following a structured approach to select, build and analyze social practices imaginaries, we “force” the identification of a diverse set of implications, derived from both the inhabitants’ and the learning system’s (and its designer’s) perspective. By taking a practice-level approach, we look beyond interactions on a user-system-level to identify these challenges and opportunities. And finally, through a future-oriented view on learning systems in an everyday domestic context, we enable proactive anticipations next to reactive reflections–looking beyond what is currently available on the market by imagining plausible futures.

## Data Availability

The original contributions presented in the study are included in the article/Supplementary Material, further inquiries can be directed to the corresponding author.
